# The neurotoxicity of amyloid β-protein oligomers is reversible in a primary neuron model

**DOI:** 10.1186/s13041-016-0284-5

**Published:** 2017-01-31

**Authors:** Daisuke Tanokashira, Naomi Mamada, Fumiko Yamamoto, Kaori Taniguchi, Akira Tamaoka, Madepalli K. Lakshmana, Wataru Araki

**Affiliations:** 1 Department of Demyelinating Disease and Aging, National Institute of Neuroscience, National Center of Neurology and Psychiatry (NCNP), Kodaira, Tokyo 187-8502 Japan; 2 Department of Neurology, Faculty of Medicine, University of Tsukuba, Tsukuba, Ibaraki 305-8575 Japan; 3 Torrey Pines Institute for Molecular Studies, Port St. Lucie, 34987-2352 Florida USA

**Keywords:** Alzheimer’s disease, Amyloid β-protein, Neurotoxicity, Oligomer, Primary neuron, Tau, β-catenin

## Abstract

Alzheimer’s disease (AD) is characterized by the accumulation of extracellular amyloid β-protein (Aβ) and intracellular hyperphosphorylated tau proteins. Recent evidence suggests that soluble Aβ oligomers elicit neurotoxicity and synaptotoxicity, including tau abnormalities, and play an initiating role in the development of AD pathology. In this study, we focused on the unclarified issue of whether the neurotoxicity of Aβ oligomers is a reversible process. Using a primary neuron culture model, we examined whether the neurotoxic effects induced by 2-day treatment with Aβ42 oligomers (Aβ-O) are reversible during a subsequent 2-day withdrawal period. Aβ-O treatment resulted in activation of caspase-3 and eIF2α, effects that were considerably attenuated following Aβ-O removal. Immunocytochemical analyses revealed that Aβ-O induced aberrant phosphorylation and caspase-mediated cleavage of tau, both of which were mostly reversed by Aβ-O removal. Furthermore, Aβ-O caused intraneuronal dislocation of β-catenin protein and a reduction in its levels, and these alterations were partially reversed upon Aβ-O withdrawal. The dislocation of β-catenin appeared to reflect synaptic disorganization. These findings indicate that removal of extracellular Aβ-O can fully or partially reverse Aβ-O-induced neurotoxic alterations in our neuron model. Accordingly, we propose that the induction of neurotoxicity by Aβ oligomers is a reversible process, which has important implications for the development of AD therapies.

**Electronic supplementary material:**

The online version of this article (doi:10.1186/s13041-016-0284-5) contains supplementary material, which is available to authorized users.

## Introduction

Alzheimer’s disease (AD) is a progressive neurodegenerative disorder characterized clinically by memory loss and cognitive decline. Its major pathological hallmarks are extracellular senile plaques and intracellular neurofibrillary tangles, which are composed of amyloid β-protein (Aβ) and phosphorylated tau (p-tau) protein, respectively [1]. A central role of Aβ in the molecular pathology of AD has been established [2]. Aβ is generated by sequential cleavages of amyloid precursor protein (APP) by β-site APP cleaving enzyme 1 (BACE1) and γ-secretase [3]. Under pathological conditions, Aβ self-aggregates to form Aβ oligomers, which likely induce abnormalities of tau and cause cellular stress responses, including caspase activation and disturbances of synaptic structure and plasticity. Thus, Aβ oligomers are considered to be an initiator of AD pathology [4–7]. The mechanisms by which Aβ oligomers induce neurotoxicity, critical issues from a therapeutic standpoint, remain to be elucidated, although several hypotheses have been suggested [4–10]. The major theory is that extracellular Aβ oligomers interact with certain cell surface receptors to cause aberrant signal transduction. Alternatively, it has been suggested that extracellular Aβ oligomers disrupt the cell membrane directly or intracellular Aβ oligomers elicit neurotoxicity. Although a link between Aβ oligomers and tau has been established [11, 12], signaling pathways linking the two remain elusive. It also remains to be clarified whether the neurotoxicity of Aβ oligomers is reversible and abates upon their removal.

We previously established a primary neuron culture model in which Aβ oligomers trigger apparent neurotoxicity with relatively modest neuronal death [13]. In the current study, we took advantage of this system to investigate the reversibility of Aβ oligomers-associated neurotoxicity, characterized by caspase activation and tau abnormalities. Here, we present evidence that the neurotoxicity of Aβ oligomers is reversible in primary neurons.

## Results

### Reversal of Aβ oligomer-induced caspase-3 and eIF2α activation upon oligomer removal

**Fig. 1:**
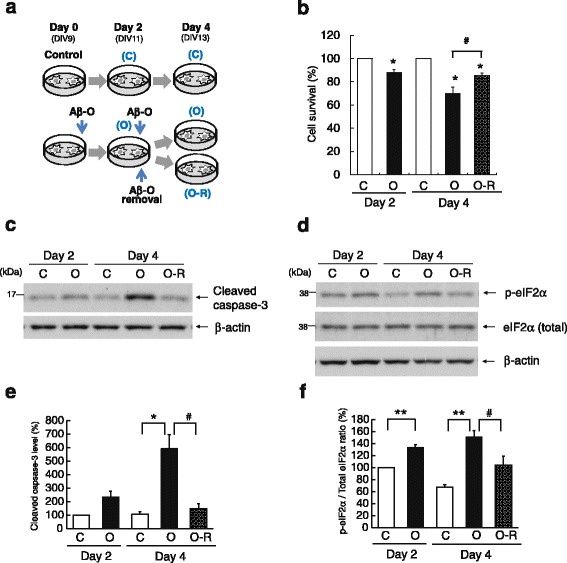
Effects of Aβ-O treatment and removal on caspase-3 and eIF2α in primary cortical neurons. **a** Experimental design. Primary neurons (DIV9) were treated with 2.5 μM Aβ-O (O) or vehicle (C) for 2 days. On day 2, Aβ-O-treated neurons were washed and further treated with Aβ-O (O) or deprived of Aβ-O (O-R) for an additional 2 days. Control neurons (C) were similarly cultured for an additional 2 days. **b** Cell survival assay. Cell survival was analyzed by CCK-8 assay as described in Methods. The graph shows survival levels relative to those in controls on day 2 or 4. **c** Cells were lysed on day 2 or 4, and cell lysates were analyzed by Western blotting using an anti-cleaved caspase-3 antibody. **d** Cleaved caspase-3 levels were quantified and expressed relative to those in control neurons on day 2. **e** Cell lysates were analyzed by Western blotting using anti-eIF2α or anti-p-eIF2α antibodies. **f** Total and p-eIF2α levels were quantified and expressed as p-eIF2α/total eIF2α ratios relative to those in control neurons on day 2. Data represent means ± SEM from three separate experiments. *p < 0.05, **p < 0.01, compared with control. #*p* < 0.05, compared with Aβ-O-treated cells

We established a primary neuron culture model in which treatment of neurons at 9 days in vitro (DIV9) with 2.5 μM Aβ42 oligomers (Aβ-O) exert neurotoxic effects with modest cell death [13]. In this model, Aβ-O induces activation of caspase-3, a major apoptosis marker, and eIF2α, a mediator of various stress responses [14]. During 3 days of Aβ-O treatment, a time-dependent increase in cleaved caspase-3 levels was observed, which corresponded to a time-dependent slight decrease in cell survival [13], suggesting progression of neuronal degeneration during the treatment period. We inferred that the capacity of neurons to recover from the Aβ-O-induced neuronal damage may be dependent on the time period of Aβ-O exposure. In our pilot experiments, neurons appeared to recover from caspase-3 activation and abnormal tau phosphorylation, the latter of which is described in the following section, upon withdrawal of Aβ from medium on day 2, whereas they did not appear to recover considerably upon Aβ removal on day 3. Therefore, we decided to investigate whether Aβ-O neurotoxicity is reversible using the experimental protocol depicted in Fig. 1a. Neurons were incubated with or without Aβ-O for 2 days, at which point cells were deprived of Aβ-O by replacing the medium with fresh medium lacking Aβ-O, or were re-provided Aβ-O, and cultured for an additional 2 days. We first estimated the effects of Aβ-O on cell survival using a Cell Counting Kit-8. Aβ-O treatment for 2 and 4 days decreased neuronal viability by 12 and 30%, respectively, compared with controls. Neuronal viability in the treatment group in which Aβ-O was removed after 2 days was ~85% of that in controls on day 4, a value significantly higher than that of neurons treated continuously with Aβ-O for 4 days (Fig. 1b).

We next investigated whether cellular stress responses to Aβ-O are reversible. Treatment of neurons with Aβ-O induced a time-dependent increase in cleaved caspase-3 levels on days 2 and 4 compared with controls (Fig. 1c, d). Cleaved caspase-3 levels on day 4 in neurons deprived of Aβ-O were significantly lower than those in neurons treated continuously with Aβ-O for 4 days, and were even lower than those on day 2 (Fig. 1c, d). Treatment of neurons with Aβ-O also significantly increased the ratio of phosphorylated to total eIF2α (p-eIF2α/total eIF2α) compared with control neurons on days 2 and 4 (Fig. 1e, f). As was the case for cleaved caspase-3 levels, the p-eIF2α/total eIF2α ratio in neurons deprived of Aβ-O was significantly decreased on day 4 compared with that in neurons treated continuously with Aβ-O for 4 days, and appeared lower than that on day 2 (Fig. 1e, f). These data indicate that removal of extracellular Aβ-O reverses Aβ-O-induced activation of caspase-3 and eIF2α.

### Aberrant tau phosphorylation induced by Aβ-O treatment is reversed upon oligomer removal

**Fig. 2:**
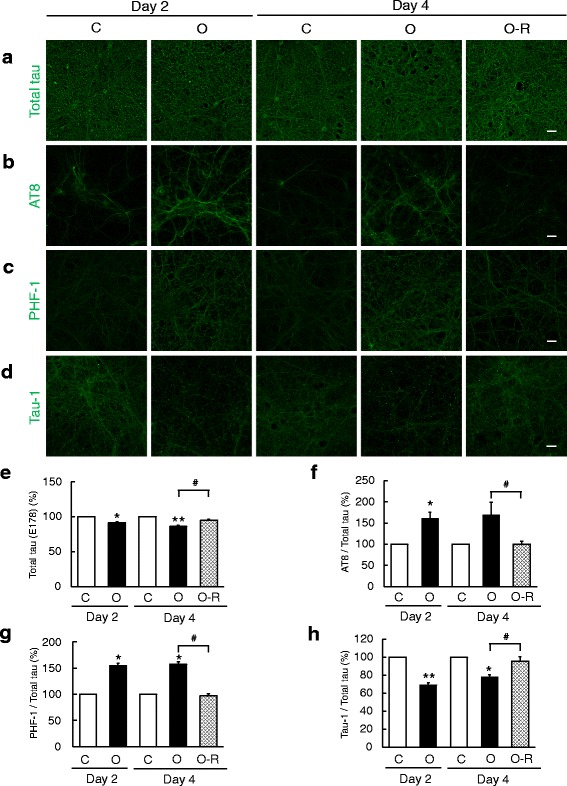
Effects of Aβ-O treatment and removal on abnormal phosphorylation of tau. Primary neurons grown on coverslips were treated as in Fig. 1, followed by immunofluorescence staining with anti-total tau (**a**), AT8 (**b**), PHF1 (**c**) or Tau-1 (**d**) antibodies. Scale bar = 20 μm. **e-h** Immunofluorescence intensities were quantified as described in Methods, and expressed relative to those in controls on day 2 or 4. For AT8 (**f**), PHF-1 (**g**), and Tau-1 (**h**), immunofluorescence intensity levels were normalized to those of total tau (**e**). Data represent means ± SEM from three separate experiments. *p < 0.05, **p < 0.01, compared with control. #*p* < 0.05, compared with Aβ-O-treated cells

Recent evidence suggests a link between Aβ-O and tau abnormalities [11, 12]. To analyze this linkage, we examined abnormal tau phosphorylation by immunocytochemistry using antibodies specific for p-tau (AT8 and PHF-1) or unphosphorylated tau (Tau-1). Total tau immunoreactivity was localized mostly in neurites (likely axons) of neurons (Fig. 2a), and was slightly decreased after 2 or 4 days of continuous exposure; this small decrease was largely eliminated in neurons deprived of Aβ-O (Fig. 2e). p-Tau was similarly located in neurites of neurons (Fig. 2b, c). Continuous Aβ-O treatment significantly increased p-tau levels in neurons, measured as the intensity of AT8 or PHF-1 signals normalized to that of total tau, on days 2 and 4 compared with that in control neurons (Fig. 2a–c, e–g). In contrast, the intensity of unphosphorylated Tau-1 immunoreactivity in neurons continuously treated with Aβ-O was significantly reduced compared with that in control neurons on days 2 and 4 (Fig. 2d, h). Notably, in neurons deprived of Aβ-O, both the intensity of p-tau immunoreactivity, revealed by AT8 and PHF-1 signals, and the intensity of unphosphorylated Tau-1 immunoreactivity, were restored to levels similar to those in control neurons on day 4 (Fig. 2b–d, f–h).

A further analysis of tau phosphorylation by Western blot analysis showed that relative levels of unphosphorylated tau (Tau-1) to total tau in Aβ-O-treated neurons decreased on days 2 and 4 compared with controls, and tended to recover upon Aβ-O withdrawal on day 4 (Additional file 1: Figure S1). Although AT8 and PHF1 unexpectedly failed to yield consistent results in Western blotting, our findings collectively suggest that Aβ-O-induced abnormalities of tau phosphorylation are reversible upon oligomer removal.

### Aβ-O-induced caspase cleavage of tau is reversed upon oligomer removal

**Fig. 3:**
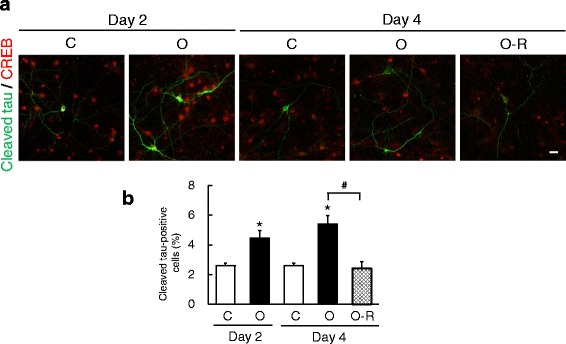
Effects of Aβ-O treatment and removal on aberrant cleavage of tau. **a** Primary neurons grown on coverslips were treated as in Fig. 1, followed by double-immunofluorescence staining with anti-cleaved tau (green) and anti-CREB (red) antibodies. Scale bar = 20 μm. **b** The percentage of all CREB-positive cells positive for cleaved tau was calculated as described in Methods and graphed. Data represent means ± SEM from three separate experiments. *p < 0.05, compared with control. #*p* < 0.05, compared with Aβ-O-treated cells

It is known that activated caspase mediates the truncating cleavage of tau [15, 16]. To investigate whether Aβ-O affects tau cleavage, we performed immunocytochemistry using an antibody specific for the caspase-cleaved form of tau. A limited number of untreated neurons were immunopositive for cleaved tau, but on days 2 and 4 following treatment with Aβ-O, the percentage of cleaved-tau-positive cells was notably increased compared with control neurons (Fig. 3a, b). Removal of Aβ-O substantially reduced the proportion of cells positive for cleaved tau, restoring it to a value similar to that in controls on day 4 (Fig. 3a, b).

### Aβ-O treatment causes abnormal alterations in β-catenin that are partially reversible upon oligomer removal

**Fig. 4:**
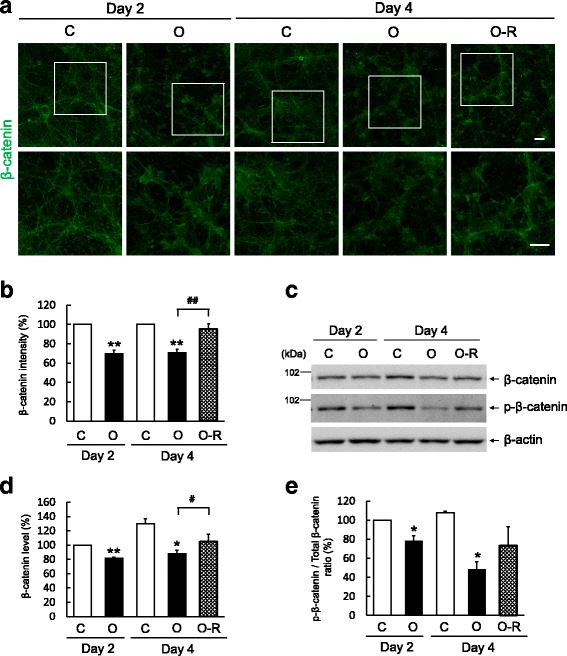
Effects of Aβ-O treatment and removal on β-catenin in primary cortical neurons. **a** Primary neurons grown on coverslips were treated as in Fig. 1, followed by immunofluorescence staining with an anti-β-catenin antibody. Bottom panel shows high-magnification images of the regions depicted by white squares in upper images. Scale bar = 20 μm. **b** Immunofluorescence intensity of β-catenin staining was quantified as described in Methods and expressed relative to levels in control neurons on day 2 or 4. Data represent means ± SEM from three separate experiments. **c** Primary neurons were treated as in Fig. 1, followed by Western blot analysis of cell lysates using the indicated antibodies. **d, e** β-catenin (**d**) levels and p-β-catenin/total β-catenin ratios (**e**) expressed relative to those in control neurons on day 2. Data represent means ± SEM from three separate experiments. *p < 0.05, **p < 0.01, compared with control. #*p* < 0.05, ##*p* < 0.01, compared with Aβ-O-treated cells

It has been reported that AD pathology is associated with disrupted Wnt/β-catenin signaling [17, 18]. β-catenin also plays important roles in the regulation of synaptic structures and plasticity [19, 20]. To investigate the involvement of β-catenin in the neurotoxic mechanism of Aβ-O, we first analyzed the intraneuronal localization of β-catenin by immunocytochemistry. β-catenin immunoreactivity was mainly observed in punctate structures over neurites of control neurons, suggesting synaptic localization, consistent with the role of β-catenin in synaptic vesicle localization and presynaptic assembly [19]. Continuous Aβ-O treatment decreased the intensity of β-catenin immunoreactivity in neurons on days 2 and 4 compared with that in controls (Fig. 4a, b). Interestingly, Aβ-O apparently induced a dramatic change in the intraneuronal localization of β-catenin; positive immunoreactivities were observed mainly in neurites and neuronal soma with markedly reduced punctate staining, implying a shift from synapses to neurites and soma. Withdrawal of Aβ-O partially reversed the decreased intensity of β-catenin immunoreactivity as well as its abnormal intraneuronal localization on day 4 compared with neurons treated continuously with Aβ-O (Fig. 4a, b). We further analyzed the protein level of β-catenin by Western blotting. β-catenin levels in control neurons increased with the age of the culture and were lower in Aβ-O-treated neurons than control neurons on days 2 and 4 (Fig. 4c, d). Upon Aβ-O withdrawal, β-catenin recovered to a level intermediate between those in control and Aβ-O-treated cells (Fig. 4c, d). In addition, continuous Aβ-O treatment induced a significant, time-dependent reduction in the phospho-β-catenin (p-β-catenin)/total β-catenin ratio compared with controls on days 2 and 4 that was partially reversed on day 4 by removal of Aβ-O (Fig. 4c, e). To examine the relationship between Aβ-O-induced β-catenin alterations and synapses, we performed double immunostaining of β-catenin and SNAP-25, a presynaptic SNARE protein [21]. We observed that both proteins underwent similar alterations in intraneuronal localization following Aβ-O treatment (Additional File 2: Figure. S2), implying that the abnormal alterations of β-catenin are associated with disorganization of synapses.

## Discussion

We have established a primary neuronal culture model in which relatively low concentrations of Aβ-O induce modest neuronal death. Our data showed that Aβ-O induces activation of caspase-3 and eIF2α, and abnormal phosphorylation and cleavage of tau. These abnormal alternations have been reported to be present in AD brains [11, 12, 14, 15, 22–24], suggesting that our model reflects the characteristic features of AD pathology. Our study also provides evidence of a direct link between Aβ-O and tau abnormalities, in accord with previous studies [25–30]. To evaluate whether Aβ-O neurotoxicity is a reversible or irreversible process, we used an experimental paradigm in which neurons exposed to Aβ-O for 2 days were further treated with Aβ-O for 2 additional days or were deprived of Aβ-O for this same culture period. We then compared control and Aβ-O–treated neurons on day 2, and control, Aβ-O-treated and Aβ-O–deprived neurons on day 4. We first focused on caspase-3 and eIF2α, both of which are thought to be important in mediating AD neurodegenerative processes [15, 24]. We found that the levels of cleaved caspase-3 and p-eIF2α in Aβ-O-deprived neurons were much lower on day 4 than those in neurons continuously treated with Aβ-O, and were similar to those in controls. These findings suggest that neurons can recover following Aβ-O removal, even after neuronal injury responses to Aβ-O have already progressed.

We examined the abnormal phosphorylation of tau by detection with the antibodies AT8 and PHF-1, which recognize major phosphorylation sites characteristic of AD [31]. Immunocytochemically, AT8 and PHF-1 signals increased whereas unphosphorylated Tau-1 immunoreactivity decreased on days 2 and 4 in neurons treated continuously with Aβ-O compared with those in control neurons. Intriguingly, Aβ-O removal reversed these alterations in AT8, PHF-1, and Tau-1 immunoreactivities. In considering possible mechanisms underlying this recovery, we note that the phosphorylation sites recognized by AT8 and PHF-1 are known to be targeted by the major tau kinases GSK3β and Cdk5 [32, 33]; thus, Aβ-O-induced abnormal tau phosphorylation may be attributable to activation of these kinases [31]. Accordingly, it is possible that Aβ-O removal may induce deactivation of these kinases, leading to dephosphorylation of tau at the corresponding target sites. In addition, it is conceivable that the activity of tau-targeted phosphatases also increases following Aβ-O withdrawal.

Our analyses further demonstrated that Aβ-O treatment increased the proportion of neurons positive for cleaved tau on days 2 and 4, and showed that this effect was fully reversed following Aβ-O deprivation for 2 days. It is likely that Aβ-O induces tau cleavage through caspase activation, since caspase-3 is known to cleave tau at Asp421 [15]. Consistent with this view, the reversal of tau cleavage paralleled with that of caspase-3 activation upon Aβ-O withdrawal. Recent studies have suggested that cleaved tau is prone to aggregate and may act as a seed for formation of aggregates [22], and that caspase-mediated cleavage of tau initiates tangle formation [34]. Moreover, cleaved tau appears to be preferentially released from neurons for possible interneuronal transmission [35, 36]. Taken together, our findings that abnormal phosphorylation and truncation of tau induced by Aβ-O were reversed upon oligomer removal suggest that these tau alternations are reversible processes. It has been suggested that Aβ oligomers induce tau missorting from axons to dendrites [37, 38]. Further studies are necessary to investigate whether such tau missorting occurs in our model system.

We found that the localization pattern of β-catenin was markedly altered by Aβ-O treatment, implying dissociation of β-catenin from synapses. Further, β-catenin levels were decreased in Aβ-O-treated neurons compared with controls. We observed that Aβ-O removal reversed these abnormal β-catenin alterations, consistent with the idea that Aβ-O–induced neuronal insults are reversible. Aβ-O-induced β-catenin dislocation may be associated with perturbation of synaptic organization. This possibility is supported by our observation that SNAP-25 was similarly dislocated upon Aβ-O treatment (Fig. S2). Consistent with this, synapsin I, a representative presynaptic protein, and αN-catenin, a binding partner of β-catenin [39], were observed to exhibit similar abnormal dislocation following Aβ-O treatment (data not shown). These observations are consistent with the notion that Aβ oligomers disrupt synaptic structures and functions in vitro and in vivo [4, 6, 7, 40].

Many reports have suggested that disturbances in Wnt/β-catenin signaling are linked to AD [17, 18]. Aβ-induced activation of apoptosis and synaptotoxicity are reported to be antagonized by certain Wnt agonists such as Wnt-3a, implying that Aβ disrupts Wnt signaling, possibly through interaction with frizzled receptors [41–45]. Since the Wnt signaling pathway negatively regulates GSK3β [46], it is plausible that treatment with Aβ oligomers inhibits Wnt/β-catenin signaling and induces activation of GSK3β. In the canonical Wnt signaling pathway, phosphorylation of β-catenin by GSK3β facilitates β-catenin degradation [17, 18]. However, our finding that p-β-catenin levels were reduced by Aβ-O exposure suggests that the decrease in β-catenin in our experimental system was independent of β-catenin phosphorylation.

**Fig. 5:**
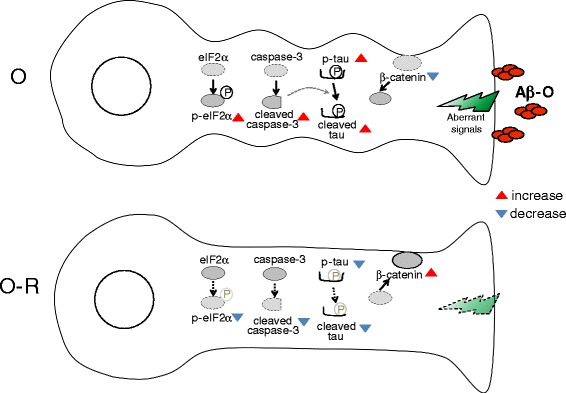
Schema illustrating a possible mechanism by which neurotoxic effects of Aβ-O are reversed by its removal. Aβ-O possibly acts on the cell surface of neurons to transmit aberrant signals, resulting in various abnormal cellular responses, including caspase-3 activation, eIF2α activation, tau phosphorylation and cleavage, and abnormal subcellular localization of β-catenin and a reduction in its levels (upper schema, O). Caspase activation is likely responsible for tau cleavage. The alterations of β-catenin may be associated with disorganization of synapses. Upon Aβ-O removal, the aberrant signals subside, resulting in reversal of all abnormal responses (lower schema, O-R)

Several hypotheses have been suggested to account for the mechanism by which Aβ oligomers induce neurotoxicity [4–10]. One major model is that extracellular Aβ oligomers interact with certain cell surface receptors, resulting in aberrant signal transduction [4–7, 10]. Alternatively, it has been suggested that Aβ oligomers disrupt the cell membrane directly or that intracellular Aβ oligomers cause neurotoxicity [8, 9]. Our demonstration that pathological alternations induced by Aβ-O can be reversed by its extracellular removal tends to favor the idea that Aβ-O neurotoxicity is mediated by certain cell-surface proteins; thus, at least under our experimental conditions, other models appear unlikely (Fig. 5).

Our results indicate that neurotoxicity induced by Aβ oligomers is a reversible process in that neurons are capable of recovering from the moderate neurotoxic insults. The present findings are consistent with a small number of prior reports. Lee et al. [47] showed that the impairment in synaptic activity induced by short-term treatment with Aβ oligomers is reversible. Brikha et al. [48] used mouse organotypic slices to show that Aβ-induced spine loss recovers following Aβ washout. An in vivo study using APP transgenic mice reported that Aβ-associated neuritic dystrophy can be recovered by application of anti-Aβ antibody [49]. More importantly, a recent study reported that cognitive dysfunction in APP transgenic mice is restored following suppression of the transgene, an effect that was attributed to a reduction in Aβ oligomers [50]. This in vivo observation appears to be consistent with our in vitro findings, and suggests that any treatment designed to remove or reduce Aβ oligomers would be effective against AD. One example of such treatments is immunotherapy targeting Aβ oligomers, which has been shown to reduce Aβ burden and improve synaptic and cognitive deficits in animal models [51, 52]. Clinical trials of passive immunotherapy using antibodies specific for Aβ oligomers are in progress [53]. In addition, the use of BACE1 inhibitors to prevent Aβ oligomer accumulation is also a promising therapeutic option [54].

In summary, we investigated the reversibility of Aβ oligomer-induced neurotoxicity, using a neuron model system which reflects the characteristic features of AD pathology. We demonstrated that Aβ-O treatment induces activation of caspase-3 and eIF2α, aberrant phosphorylation and caspase-mediated cleavage of tau, and abnormal alterations in β-catenin, and showed that all of these abnormalities were fully or partially reversed upon extracellular removal of Aβ-O. These findings collectively suggest that Aβ oligomers-associated neurotoxicity is a reversible process, supporting the view that treatments targeting Aβ oligomers have significant therapeutic potential for AD. Further studies on the molecular mechanisms underlying the reversibility of Aβ oligomers-induced neurotoxicity will contribute to the development of novel therapeutic strategies to treat or prevent AD.

## Methods

### Cell culture

Primary neuronal cultures were prepared from cerebral cortices of rat embryos at embryonic day 17, essentially as described previously [13, 55]. Neurons were plated on poly-L-lysine–coated plates or dishes at a density of 680 cells/mm^2^. Cells were maintained in a humidified atmosphere of 5% CO_2_/95% air in Macs Neuro Medium (Miltenyi Biotec, Auburn, CA, USA) containing 0.5 mM L-glutamine, NeuroBrew-21 (Miltenyi Biotec), and penicillin-streptomycin. Half of the medium was replaced with fresh medium every 3–4 days.

### Aβ preparation and treatment

Aβ42 oligomers (Aβ-O) were prepared as described previously [13, 56]. Briefly, human Aβ(1–42) peptide (Peptide Institute, Osaka, Japan) was dissolved in 1,1,1,3,3,3-hexafluoro-2-propanol (HFIP; Sigma, St Louis, MO, USA) in a chemical fume hood to obtain a 1 mM solution. HFIP was evaporated overnight in the hood and further under vacuum for 1 h, and dried peptide films were stored at –30 °C. Prior to use, a 5 mM stock was prepared by dissolving dried Aβ peptide in dimethyl sulfoxide (DMSO), and sonicating it in an ultrasonic bath sonicator for 10 min. Oligomers were prepared by diluting 5 mM Aβ DMSO stock to 0.1 mM with DMEM/F12 without phenol red and left for 1 day at 4 °C. Immediately before addition to DIV9 neurons, Aβ-O preparations were diluted to 2.5 μM with neuronal medium and used to replace the entire medium. Control cultures were treated with the same concentration of DMSO [13]. After Aβ-O treatment for 2 days, the cells were rinsed twice and incubated with fresh medium with or without Aβ-O for an additional 2 days (Fig. 1a).

### Antibodies

The antibodies used were as follows: anti-cleaved caspase-3 (Asp175) (Cell Signaling, Danvers, MA, USA); anti-eIF2α (Assay Biotechnology, Sunnyvale, CA, USA); anti-p-eIF2α (Ser51) (Cell Signaling); AT8, which recognizes tau protein phosphorylated at Ser202 and Thr205 (Thermo Scientific, Rockford, IL, USA); PHF-1, which recognizes tau phosphorylated at Ser396 and Ser404 [57] (provided by Dr. Peter Davies); Tau-1, which recognizes unphosphorylated epitopes between residues 192 and 204 (Millipore, Darmstadt, Germany); anti-cleaved tau (Asp421) (Millipore); anti-total tau (E178, Abcam, Cambridge, MA, USA; Tau-5, Thermo Scientific); anti-CREB (Gene Tex, Irvine, CA, USA); anti-β-catenin (Cell Signaling); anti-p-β-catenin (Ser33 + Ser37) (Abcam); anti-SNAP-25 (BioLegend, San Diego, CA, USA) and anti-β-actin (Wako, Osaka, Japan).

### Western blot analysis

Cells were lysed in radioimmunoprecipitation assay (RIPA) buffer containing protease inhibitors and phosphatase inhibitors, and cell lysates were prepared as described previously [13]. For Western analysis of tau, cells were lysed in sodium dodecyl sulfate (SDS) lysis buffer (62.5 mM Tris pH 6.8, 2% SDS, 10% glycerol, 1 mM dithiothreitol, 5 mM EDTA) containing protease inhibitors and phosphatase inhibitor cocktail, and heated at 95 °C for 10 min, followed by centrifugation at 100,000 g for 30 min. Western blotting of cell lysates was performed using a standard procedure, as described previously [13]. Protein band densities were quantified using an LAS-1000 image analyzer (Fuji Film Co., Tokyo, Japan).

### Cell survival assay

Cell survival assays were performed as described previously [13]. Briefly, primary cortical neurons cultured on 12-well plates were treated with Aβ-O or vehicle, as described above. Cell Counting Kit-8 solution (Dojindo, Kumamoto, Japan) was added to each well, and the plates were left in a CO_2_ incubator for 2 h. Absorbance was measured at 450 nm using a microplate reader. Absorbance of the medium, used as a blank, was subtracted from that of each sample.

### Immunocytochemistry

Immunocytochemical analyses were performed as described previously [13, 58]. Briefly, primary neurons cultured on coverslips were fixed with 4% paraformaldehyde in phosphate-buffered saline (PBS). Fixed cells were permeabilized and blocked with 0.3% Triton X-100 and 1% horse serum in PBS, and incubated with primary antibody for 1 h, followed by incubation with Alexa488-conjugated anti-mouse or anti-rabbit IgG secondary antibodies (Molecular Probes, Eugene, OR, USA) for 1 h. For double-immunofluorescence staining, cells were subsequently incubated with another primary antibody and Alexa568-conjugated specific secondary antibody. Specimens were observed under an LSM 780 laser-scanning confocal microscope (Carl Zeiss, Jena, Germany). Images illustrating antibody labeling were acquired using a 20× 0.8 N.A. dry objective. The mean fluorescence intensity of tau (E178), AT8, PHF-1 and Tau-1 was quantified using the entire image (212.55 × 212.55 μm) as the region of interest. In every immunostaining experiment, 4–5 images were analyzed for each condition. The values for AT8, PHF-1 and Tau-1 were normalized to that of tau (E178). Cleaved tau was analyzed in cells doubly immunostained with antibodies against cleaved tau and CREB, the latter of which was used to visualize viable neurons. The percentage of CREB-positive neurons positive for cleaved tau in the whole image was calculated. Five images were analyzed for each condition in every experiment.

### Statistical analysis

All results are presented as means ± SEMs. Data were statistically analyzed using one-way analysis of variance (ANOVA) followed by a Tukey multiple comparison test or Student’s *t*-test with a significance threshold of *p* < 0.05.

## Additional files

Additional file 1: Figure S1. Western blot analysis of tau phosphorylation. Primary neurons treated as in Fig. 1 were lysed in SDS lysis buffer. Cell lysates were analyzed by Western blotting using anti-total tau (Tau-5) and Tau-1 antibodies. Tau-1/Tau-5 ratios were expressed relative to those in control neurons on day 2 or 4. Data represent means ± SEM from three separate experiments. **p* < 0.05, compared with control. #*p* < 0.05, compared with Aβ-O-treated cells. (PDF 1333 kb)
Additional file 2: Figure S2. The relationship between Aβ-O-induced alterations of β-catenin and synapses. Primary neurons treated as in Fig. 1 were doubly immunostained with anti-β-catenin (green) and anti-SNAP-25 (red). Similar alterations in intraneuronal localization of both proteins were observed following Aβ-O treatment and removal. Evident co-localization was not observable between the two proteins. (PDF 548 kb)

## Abbreviations

AD: Alzheimer’s disease
Aβ: Amyloid β-protein
Aβ-O: Aβ42 oligomers
DIV: Days in vitro
PBS: Phosphate-buffered saline
p-tau: Phosphorylated tau.
