# Supplementary figures and images for: The neurotoxicity of amyloid β-protein oligomers is reversible in a primary neuron model
Source: Mol Brain. 2017 Jan 31;10:4. doi: 10.1186/s13041-016-0284-5 (PMC5282621; doi:10.1186/s13041-016-0284-5)

Additional file 1: Fig. S1

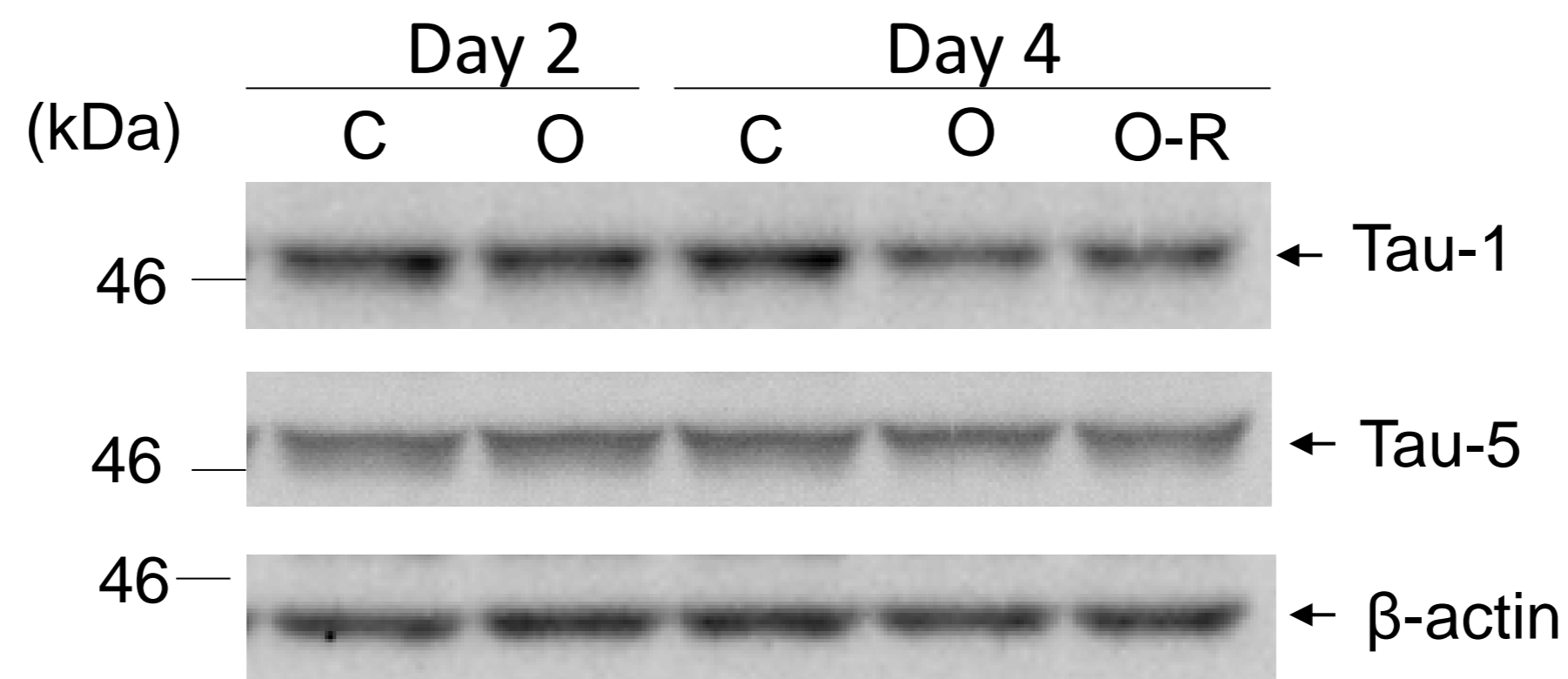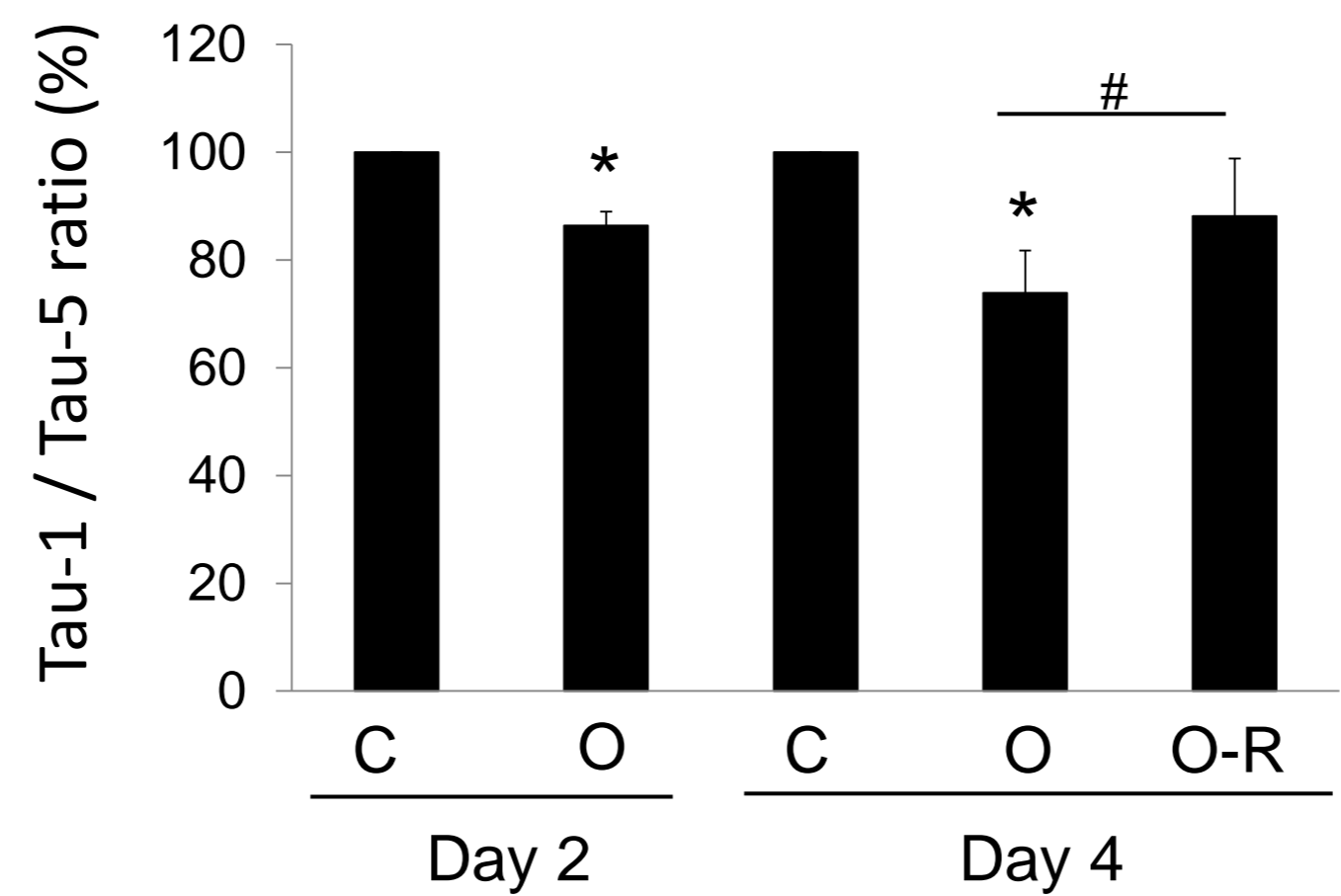

Supplement: Additional file 1: Figure S1. — Western blot analysis of tau phosphorylation. Primary neurons treated as in Fig. 1 were lysed in SDS lysis buffer. Cell lysates were analyzed by Western blotting using anti-total tau (Tau-5) and Tau-1 antibodies. Tau-1/Tau-5 ratios were expressed relative to those in control neurons on day 2 or 4. Data represent means ± SEM from three separate experiments. *p < 0.05, compared with control. #p < 0.05, compared with Aβ-O-treated cells. (PDF 1333 kb) [file 13041_2016_284_MOESM1_ESM.pdf]

## Additional file 2: Fig. S2

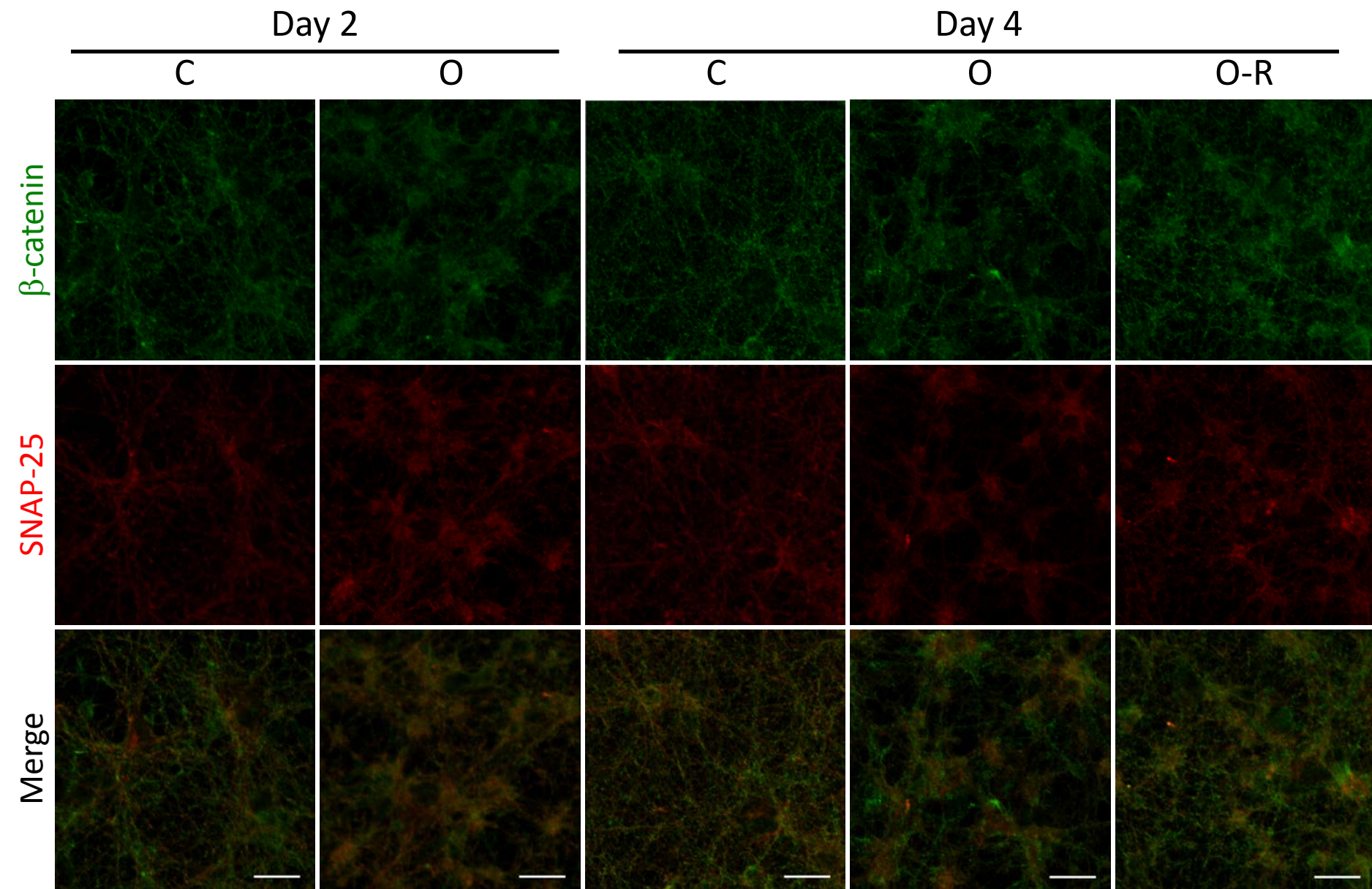

Supplement: Additional file 2: Figure S2. — The relationship between Aβ-O-induced alterations of β-catenin and synapses. Primary neurons treated as in Fig. 1 were doubly immunostained with anti-β-catenin (green) and anti-SNAP-25 (red). Similar alterations in intraneuronal localization of both proteins were observed following Aβ-O treatment and removal. Evident co-localization was not observable between the two proteins. (PDF 548 kb) [file 13041_2016_284_MOESM2_ESM.pdf]
